# E-Cadherin in Pancreatic Ductal Adenocarcinoma: A Multifaceted Actor during EMT

**DOI:** 10.3390/cells9041040

**Published:** 2020-04-22

**Authors:** Michele Sommariva, Nicoletta Gagliano

**Affiliations:** Department of Biomedical Sciences for Health, Università degli Studi di Milano, 20133 Milan, Italy; michele.sommariva@unimi.it

**Keywords:** E-cadherin, epithelial-to-mesenchymal transition, pancreatic adenocarcinoma

## Abstract

Epithelial-to-mesenchymal transition (EMT) is a step-wise process observed in normal and tumor cells leading to a switch from epithelial to mesenchymal phenotype. In tumors, EMT provides cancer cells with a metastatic phenotype characterized by E-cadherin down-regulation, cytoskeleton reorganization, motile and invasive potential. E-cadherin down-regulation is known as a key event during EMT. However, E-cadherin expression can be influenced by the different experimental settings and environmental stimuli so that the paradigm of EMT based on the loss of E-cadherin determining tumor cell behavior and fate often becomes an open question. In this review, we aimed at focusing on some critical points in order to improve the knowledge of the dynamic role of epithelial cells plasticity in EMT and, specifically, address the role of E-cadherin as a marker for the EMT axis.

## 1. Introduction

The Italian anatomist Angelo Ruffini (1864–1929) demonstrated the close relationship between function and morphology, showing that biological structures exhibit a characteristic morphology suitable to play a biological role. Accordingly, the cuboidal-columnar shape of epithelial cells is strategic for their arrangement to build lining epithelia constituted by apposed cells. As a consequence, if they change their epithelial into fibroblast-like morphology, also their biological and functional properties become different. Epithelial-to-mesenchymal transition (EMT) is a process that provides cancer cells with a metastatic phenotype characterized by the loss of the epithelial phenotype and E-cadherin down-regulation, playing a key role in the progression, metastasis and chemoresistance. EMT was observed in different tumor histotypes, such as ovarian, breast, kidney and lungs as well as pancreatic ductal adenocarcinoma (PDAC) [1].

PDAC represents the fourth most common cause of cancer death in the Western world, with an estimated incidence of more than 40,000 cases per year in the United States and 448,000 cases globally. It is one of the most devastating, aggressive and lethal tumors, characterized by a 5-year survival for all stages of the disease <7% [2,3,4], due to the high incidence of recurrence and metastases dissemination [5].

In this review, we emphasize the issues related to the analysis of E-cadherin expression relative to phenotypic changes in PDAC cells during EMT. We aim to focus critical points in order to improve the knowledge of the dynamic role of epithelial cells plasticity in EMT and, specifically, address the role of E-cadherin as a marker for the EMT axis.

For this purpose, we analyze how E-cadherin expression can be influenced by the different experimental settings and environment to better understand how 3D arrangement or extracellular matrix (ECM) components occurring in the tumor microenvironment could affect its expression during EMT.

## 2. Epithelial-to-Mesenchymal Transition

**Figure 1:**
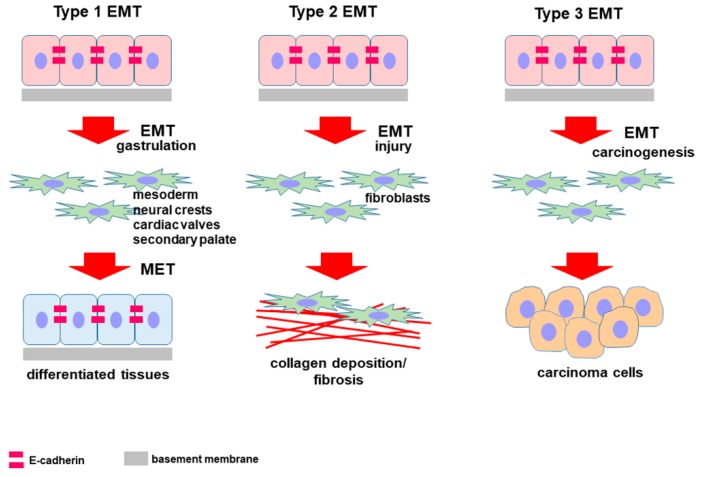
Diagram showing the classification of epithelial-to-mesenchymal transition (EMT) and the different fate of cells undergoing the EMT program. Type 1 EMT occurs in physiological conditions such as embryogenesis, Type 2 EMT can be described in pathological situations such as fibrogenesis, while Type 3 is a key event in carcinoma progression. Independently of the specific condition, the different EMT types share a common characteristic: They originate from very motile mesenchymal cells. The diverge for the control and duration of the process but, above all, for the final destination of cells generated by EMT.

EMT is an example of epithelial cell plasticity since it is a process consisting in a series of events that convert epithelial cells into mesenchymal cells [6,7,8]. To date, three types of EMT have been described, all of them generate mesenchymal fibroblast-like cells having a similar phenotype but a different final destination (Figure 1). Type 1 EMT occurs during embryonic development as part of gastrulation to form tissues and organs, Type 2 can be observed in adult tissues in response to injury or inflammation and leading to fibrosis, while Type 3 is a part of the metastatic process of carcinoma [1,9,10,11].

The morphologic and structural characteristic of epithelial cells is pivotal for their organization into tightly bound layers displaying a regular cuboidal/columnar morphology with functionally distinct apical and basolateral plasma membrane domains. Epithelial cells lay on a basement membrane, are apposed, connected by abundant cell junctions including occluding and anchoring junctions. Indeed, epithelial cells exhibit a specific “proteome” based on the expression of E-cadherin, ZO-1, claudins, occludins and cytokeratins.

**Figure 2:**
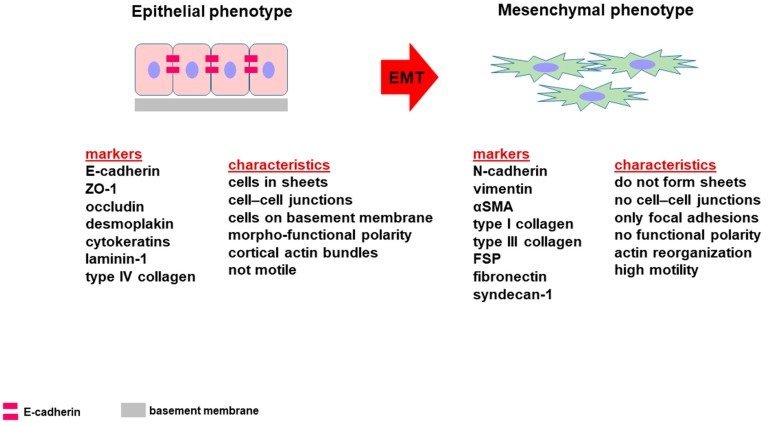
Diagram showing the morpho-functional phenotypic characteristics of epithelial and mesenchymal cells. The “proteome” related to EMT is based on the loss of some epithelial markers and the acquisition of a mesenchymal phenotype.

By contrast, mesenchymal cells exhibit an elongated fibroblastoid morphology, they are not polarized with leading-edge/trailing-edge asymmetry and do not form cell junctions but only transient adhesions to neighboring cells and focal adhesion to the ECM. Furthermore, they are characterized by high motility and migration capability and express mesenchymal markers such as N-cadherin, vimentin, α-smooth muscle actin and collagen type I (Figure 2).

During EMT, the acquisition of a “mesenchymal” phenotype by epithelial cells is allowed by the down-regulation of epithelial markers and the up-regulation of mesenchymal markers [12,13]. The decreased expression of E-cadherin paralleled with an increased expression of N-cadherin, the so-called “cadherin switch”, was described in different carcinomas, including PDAC [14,15,16].

N-cadherin is considered as an indicator of ongoing EMT and its expression was detected during the development of various types of carcinoma [16,17,18,19,20]. N-cadherin expression was detected in 13 of 30 primary pancreatic cancers, and in 8 of 15 metastatic tumors. In primary tumors, its expression correlated with neural invasion and the histological type, while in metastatic tumors it was associated with the expression of vimentin, which was lower in the primary tumor, but was significantly increased in liver metastases [14]. N-cadherin was similarly expressed at the interface between tumor and adjacent normal tissue representing the “leading edge” and the central regions of PDAC analyzed by tissue microarray [21]. It plays key roles in PDAC cells survival, invasion and metastasis [22,23,24].

## 3. E-Cadherin and EMT

**Figure 3:**
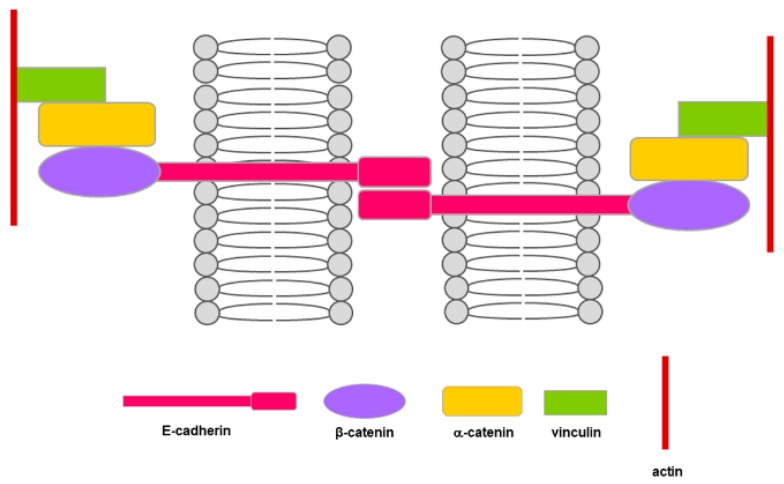
Schematic representation of E-cadherin and its relationship with the components forming adherens junctions. The extracellular domain of the E-cadherin transmembrane proteins mediates the cell–cell anchoring, while its cytoplasmic domain interacts with proteins that bridge E-cadherin with the supporting microfilaments.

E-cadherin is a member of the cadherins superfamily. It is a transmembrane protein having a 120 kDa molecular weight. Its cytoplasmic tail is associated to various catenins (α, β and p120) (Figure 3) that mediate the link with the actin cytoskeleton [25].

E-cadherin is required for the formation of stable and functional adherens junctions and acts as the major determinant of the epithelial phenotype by influencing cell polarity and tissue integrity. Moreover, a role for E-cadherin in contact inhibition was demonstrated in vitro [26,27]. However, when considering E-cadherin biological and functional properties, it is intriguing also to refer to the involvement of adherens junctions as agents in cell mechanics due to their interactions with actomyosin cytoskeleton, so that they can operate an integration between adhesion and contractility, thus affecting cellular activities [28].

E-cadherin loss is described as a key event of EMT both in vivo and in some cancer cell lines, including lung, breast, colorectal and ovarian cancer [10,29,30]. Its downregulation, leading to decreased cell–cell adhesion, permits the separation of individual cells from the primary tumor mass, and therefore represents an important characteristic in carcinoma progression. E-cadherin downregulation is often related to the invasive potential and undifferentiated phenotype of tumor cells, and it is closely related to invasion and metastasis of early tumor cells [15,31,32,33].

The loss of E-cadherin can be the result of different mechanisms such as transcriptional repression, epigenetic silencing, mutations, endocytosis and also proteolytic cleavage [34]. E-cadherin downregulation is modulated by a complex network of signaling pathways and transcription factors, such as Slug and Snail, that were recently reviewed [16].

In normal cells, E-cadherin degradation occurs by endocytic internalization and further degradation by the proteasome or lysosome [35]. In contrast, proteolytic cleavage at the cell membrane by metalloproteinases [36] or γ-secretases [37] was described in cancer cells.

The cleavage of E-cadherin can be mediated by different enzymes and generates protein fragments that possess distinct functional properties [38]. α-secretase cleaves the protein at the extracellular face of the plasma membrane and it is catalyzed by matrix metalloproteinases (MMP-3, MMP-7, MMP-9 and MT1-MMP) [39,40,41], A-disintegrin-and-metalloproteinases (ADAM10 and ADAM15), plasmin and kallikrein 7. The result of this proteolytic cleavage breaks down the mature full-length 120-kDa E-cadherin into an extracellular N-terminal 80-kDa fragment and an intracellular C-terminal 38-kDa fragment. The ectodomain fragment, defined soluble E-cadherin, is released from the plasma membrane and diffuses acting as a signaling molecule and affecting diverse cell activities, including the upregulation of MMP-2 and MMP-9 that degrade the basement membrane favoring tumor invasion. These effects can be mediated by the activation of EGFR pathway signaling [38].

The intracellular C-terminal fragment (E-cad/CTF1), embedded within the plasma membrane, can be further cleaved by presenilin-1/2, components of γ-secretase. The result is the release of an intracellular 33-kDa fragment (E-cad/CTF2) that can be cleaved by caspase-3 to form a 29-kDa fragment (E-cad/CTF3), possibly acting as signaling molecules [34,37,40,41,42,43] and playing several roles in cells, including junction disruption, cell migration and invasion. It was reported that these effects are mediated by the activation of Wnt/b-catenin pathway signaling [36,38]. Indeed, the stimulation of cell invasion exerted by soluble E-cadherin fragments was demonstrated in ovarian carcinoma [44], MCDK [45,46] and lung cancer [47], as well as in pancreatic cancer [48]. E-cadherin fragments were detected in PDAC cells, showing a higher expression in 2D-monolayers compared to 3D-spheroids [49].

## 4. PDAC and E-Cad Expression

Although E-cadherin downregulation was described as a major requirement in aggressive and invasive carcinomas, several studies have documented invasive and aggressive tumors that maintain E-cadherin expression, and the analysis of tumor samples from multiple cancers revealed an unexpectedly high frequency of E-cadherin–positive tumors [50]. High level of E-cadherin expression was demonstrated in various invasive and metastatic cancers such as prostate cancer [51], ovarian cancer [52] and glioblastoma [53], suggesting that in certain tumors E-cadherin promotes metastasis instead of suppressing tumor progression.

The patterns of loss of E-cadherin were analyzed by immunohistochemistry in tissue microarrays of 329 surgically resected pancreatic ductal adenocarcinomas. Partial or complete loss of E-cadherin expression was detected in the 43% patients, and E-cadherin decreased expression was correlated with poor outcome [54]. However, many of the analyzed samples exhibited focal areas showing E-cadherin loss in only a subset of the neoplastic cells [54], and its expression in cancer cells was characterized by patterns with variable degrees of membrane and cytoplasmic staining in PDAC commercial cell lines [29].

The variable expression of E-cadherin in human pancreatic cancers was confirmed in a study showing that in some samples E-cadherin was lost, while in others was expressed displaying a membranous labeling pattern. These last pancreatic cancers are cohesive lesions and exhibited a differentiated phenotype, while in non-cohesive and undifferentiated foci E-cadherin resulted lost [55].

The loss of E-cadherin was described in several studies, showing that E-cadherin down-regulation in pancreatic and other cancers is associated with poor outcome. Indeed, PDACs lacking E-cadherin are poorly differentiated [15,33,56], and characterized by a poor outcome [55,57]. However, some aggressive carcinomas retain features of differentiated epithelial cells, including E-cadherin expression, that was observed at the cell membrane in 50%–70% of the PDAC samples [58].

In vitro studies showed that six out of seven PDAC commercial cell lines retain E-cadherin expression on the cell membrane, and that the expression of some EMT markers in PDAC are more similar than in benign pancreatic ducts [59,60], while its expression was undetectable in the most undifferentiated cell lines [29]. High levels of expression of E-cadherin and functional adherens cell junctions were shown in cultured PDAC cells [49,61], although they exhibited a highly invasive and metastatic potential [62]. These experimental evidences increase the importance of studies aimed at definitively clarifying the role of E-cadherin and EMT in PDAC development and progression.

## 5. Effect of 3D Arrangement on E-Cadherin Expression

Growing evidence suggests that 3D cell cultures allow one to mirror the functions of living tissues since they better mimics the physiological condition allowing cells to retain in vitro the characteristic as in the original tumor. In fact, it was suggested that 3D arrangement encodes for some key information to influence cell behavior, and it was demonstrated that 3D cell cultures mimic the 3D structure of the native tissue, therefore retaining some keys to the information encoded in the tissue architecture and reproducing the spatial, morphological, biochemical and mechanical environment [63,64,65,66]. Data obtained in 3D cell cultures suggest that E-cadherin resulted in being up-regulated in PDAC cells [49]. The intrinsic 3D architecture exerts strong effects on cell morphology and phenotype, possibly influencing the expression of E-cadherin in PDAC, and a number of studies focused on the characterization of PDAC cells phenotype in relation to the expression of E-cadherin in 3D cell culture systems.

Zeeberg and coauthors described the impact of 2D and 3D tissue cultures in affecting basal growth and morphology of a panel of PDAC cell lines, and analyzed their results after orthotopically implanting the same cell lines in mice [67]. For this purpose, PANC-1 cells, exhibiting a more mesenchymal and very aggressive phenotype, were studied using different 3D cell culture models, including concave microwells, Matrigel inclusion and the organotypic system. E-cadherin resulted significantly up-regulated in all 3D cell cultures compared to 2D monolayers [67].

An increasing number of studies demonstrated that 3D cell cultures in 3D-spheroids offer the possibility to investigate tumor cell biology by mimicking and recapitulating the in vivo situation based on three-dimensional cell architecture [64,65,68,69]. PDAC cells grown in organotypic 3D culture models were studied to investigate the effect of new therapies [70] and to evaluate the effect of drugs and drug combinations [71]. The full spectrum of tumor progression was characterized in 3D organoids, derived from either biopsies or surgically resected human pancreatic tumor specimens after orthotopic transplantation [72].

Studies using 3D cell cultures allowed us to detect key phenotypic differences in PDAC contributing to better understand their behavior and, therefore, to contribute to the identification of new therapeutic tools for PDAC. A study from our group characterized the key EMT markers in PDAC cells grown in 3D-spheroids [49], focusing on E-cadherin expression analyzed in HPAF-III, HPAC and PL45 PDAC cells cultured either in the 2D-monolayer or in 3D-spheroids. Although E-cadherin resulted expressed at the cell boundaries on the plasma membrane at a similar extent in 2D-monolayers and in 3D-spheroids, Western blot analysis showed that the full length E-cadherin was increased in in lysates obtained from 3D-spheroids compared to 2D-monolayers, consistent with stronger cell adhesion [49].

## 6. Effect of ECM on E-Cadherin Expression

The tumor microenvironment contains stromal cells, including fibroblasts, inflammatory and pancreatic stellate cells, and ECM components such as type I, IV and V collagen, fibronectin, laminin, matrix metalloproteinases (MMPs) and their inhibitors (TIMPs) and transforming growth factor-β1 [73]. ECM components play an important role as key modulators of cancer cell phenotype, especially in PDAC that is characterized by an intense desmoplastic reaction [63,74].

Among other cancers, PDAC exhibits the densest desmoplastic stroma, which can account for up to 90% of the total tumor volume, exhibiting a strong interplay between tumor cells and the surrounding stroma [75]. Type I collagen (COL-I) is the most abundant ECM component in the in pancreatic desmoplastic stroma. It was suggested that it favors integrin-mediated cell–cell adhesion, as well as proliferation and migration of PDAC cells [76]. Moreover, laminin is able to favor cancer cell growth and to affect the cytotoxicity of anticancer drugs [77].

It was recently demonstrated by immunofluorescence analysis that three PDAC cell lines, having a well-differentiated epithelial phenotype, strongly express E-cadherin at cell boundaries when grown on substrates constituted by COL or laminin or fibronectin. Interestingly, Western blot analysis revealed that E-cadherin was significantly induced when one of these cell lines (PL45) was grown on COL [78], thus suggesting that this ECM component exerts an important effect on PDAC cells phenotype. One of the mechanisms responsible for the effect of COL in modifying cell activity is based on a signal mediated by the α2β1 integrin through the focal adhesion kinase and is transduced by the discoidin domain receptor [79].

Cell–ECM cross-talk in the PDAC desmoplastic environment influences EMT also supporting the mesenchymal phenotype. It was shown that collagen adhesion promotes EMT of PDAC cells by inducing N-cadherin expression and metastasis in a murine model [79,80], and COL-I expression was detectable in cells cultured on laminin and fibronectin and, to a lesser extent, on COL-I used as a substrate [78].

## 7. EMT-Related Phenotypes and Hybrid Phenotypes

EMT during embryology consists in a series of events leading to the complete conversion of epithelial cells into mesenchymal cells. Conversely, cancer cells often can undergo an incomplete or partial EMT, exhibiting both epithelial and mesenchymal markers [8,10,81,82]. The hybrid state was described in breast, ovarian, lung, colorectal cancer, prostate cancer and renal cancer cell lines in vitro [83,84,85,86].

A study of our group analyzed the phenotype of PDAC cells grown in 3D-spheroids. The analysis of the key EMT markers was studied in HPAF-II, HPAC and PL-45 cells having an epithelial-like and well differentiated phenotype [49]. In fact, PDAC cells retained E-cadherin intensely expressed at cell boundaries, but concomitantly expressed sporadic N-cadherin at the plasma membrane, suggesting that PDAC cells experienced the typical “cadherin switch” described during EMT. Moreover, the simultaneous expression of mesenchymal markers such as αSMA was detected, and some cells expressed also collagen type I, suggesting that PDAC cells underwent EMT although an intense E-cadherin expression was retained at cell boundaries. These findings support the hypothesis that these PDAC cells exhibit a hybrid EMT-related phenotype. Partial EMT was also recently demonstrated in PDAC cells [82]. A recent interesting study analyzed cadherin expression during carcinogenesis from Pancreatic Intraepithelial Neoplasia 1 (PanIN-1) to PDAC. Although a small decrease in the staining intensity was observed between PanIN-2 and PDAC, E-cadherin was found at the sites of cell–cell contacts and remained associated with the cell membrane [87].

Since in PDAC the observation of a cadherin subtype-switching from E-cadherin to another different cadherin [14,58] was suggested as phenomenon contributing to metastatic dissemination of tumor cells [24], in this study also P-cadherin was analyzed. In fact, vitro studies demonstrated that P-cadherin induces pancreatic tumor cell motility and invasiveness [88,89]. P-cadherin resulted in being expressed in the cytoplasm of healthy human pancreas, while progressively increasing at the plasma membrane during carcinogenesis starting from PanIN-1 to PDAC [87]. A coexpression and colocalization of E-cadherin and P-cadherin were observed at the cell membrane. This finding confirmed the hypothesis that cadherin subtype switching is not a general characteristic in PDAC, and that in the EMT-related phenotype both cadherins can be expressed. Both E- and P-cadherin mRNA level were detected at the same time in a high number of human samples, confirming that PDAC can exhibit a hybrid EMT-related phenotype [87].

Since PDAC can undergo an incomplete EMT, both epithelial and mesenchymal markers can be detected at a variable extent in cancer cells so that some heterogeneity in the tumor can be observed. This suggestion was demonstrated in a mouse experimental model allowing one to detect a variable degree of E-cadherin loss in PDAC. In fact, membranous localization of E-cadherin was evident in clusters of cells in well-differentiated tumor regions, while the immunoreactivity was lost in individual tumor cells in poorly differentiated areas within the same tumor [90].

The intermediate status between epithelial and mesenchymal phenotypes was defined as the “hybrid epithelial–mesenchymal (hybrid E/M)” EMT. It is regulated at the transcriptional and epigenetic level through a coordinated activity played by EMT transcription factors, such as Snail, Zeb and Twist-1, and epigenetic modulators inducing in cancer cells a dynamic plastic status that favors cancer metastasis [91]. Additionally microRNA can exert a modulation of EMT and affect E-cadherin expression in pancreatic cancer, especially miR-200 family [92], miRNA-99a [93] and miR-300 [94].

## 8. E-Cadherin and the Immune System

As already mentioned, EMT is a process that leads to profound phenotypic changes in cancer cell [95]. Especially for pancreatic cancer, characterized by a highly desmoplastic and very complex stroma [96], tumor microenvironmental stimuli play a key role in promoting and mediating EMT, cancer progression and metastatization [97]. These tumor-supporting signals can derive by different cellular types and, among them, by cells of the immune system infiltrating the pancreatic tumor mass [98]. All the mechanisms underlying how immune cells can assist tumor cell during EMT have been already extensively reviewed elsewhere [99,100]. For instance, there is a general consensus about the direct participation of tumor-associated macrophages (TAMs) in supporting EMT in pancreatic cancer. Using different cell culture medium, Meng et al. described a bidirectional cross-talk between pancreatic cancer cell and macrophages. Tumor cells influence macrophage polarization and, in turn, macrophages promote the invasiveness potential of cancer cells and modulate the expression of genes related to stemness, angiogenesis and EMT, such as E-cadherin down-modulation [101]. These observations were also confirmed by the work of Kuwada et al. that also demonstrated that macrophages could confer chemoresistance to pancreatic tumor cells, exacerbating the outcome of the disease [102]. The EMT-promoting role of macrophages can be partially explained by IL-10 production by TAMs following Toll-like receptor 4 (TLR4) activation [103]. In addition, neutrophils can also participate in PDAC EMT by releasing elastase, an enzyme able to cleave E-cadherin expressed by cancer cells [104].

Beyond the expression on epithelial and tumor cells, it is becoming clear that E-cadherin can be also expressed by cells of the immune system. E-cadherin is not generally expressed by mature T cells [105] but it can be present during their maturation process in the thymus at the stage of double positive (CD4^+^CD8^+^) thymocytes [106,107]. However, in particular circumstances, E-cadherin is reported to be expressed also by different subsets of mature T, B and NK cells, and by monocytes [108,109]. Moreover, several studies detected E-cadherin also in different subsets of dendritic cells (DCs), such as conventional DCs and Langerhans cells, and macrophages [110]. From a functional point of view, the expression of E-cadherin on DCs and macrophages seems to be involved in finely tuning the functions of these immune cells: regulating the immunogenic/tolerogenic phenotype of DCs or the proinflammatory/anti-inflammatory properties of macrophages [110]. For instance, Siddiqui et al. identified a subset of E-cadherin^+^ DCs that promote T-cell driven intestinal inflammation, indicating that this specific population of DCs possesses proinflammatory properties [111]. Accordingly, E-cadherin^+^ DCs, induced by treatment with anti-CD40 agonistic antibodies, stimulate Th_1_ and Th_17_ development and, at the same time, inhibit regulatory T cell (T_regs_) and Th_2_-based responses, eventually promoting a potent antitumor immunity [112]. On the other hand, Jiang et al. proposed a different role for E-cadherin in DCs. Based on previously published observations describing the ability of bone-marrow-derived dendritic cells (BMDMs) to form clusters whose integrity is maintained by E-cadherin [113,114,115], they found that the disruption E-cadherin DC–DC interaction determines DC activation characterized by the up-modulation of costimulatory molecules and chemokines receptors and the MHC-II re-distribution on the cell membrane. However, these DCs do not produce proinflammatory cytokines and failed to sustain an immune response. On the contrary, they promote the generation of T_regs_ [116]. Accordingly, it was found that in DCs β-catenin, an important transcription factor downstream E-cadherin signaling pathway [117], is essential for the expression of immunosuppressive mediators and for the stimulation of T_regs_ and the concomitant suppression of effector T cells [118]. Moreover, it has also been proposed that dendritic cells may contribute to sustaining an immunosuppressive tumor microenvironment after up-regulation of E-cadherin on their membrane, a phenomenon triggered by soluble factors secreted by tumor cells [119]. All these data suggest that E-cadherin is important in mediating a tolerogenic phenotype in DCs.

E-cadherin expression is also reported in macrophages, immune cells with a high degree of plasticity [110]. Generally speaking, macrophages can be divided in two big groups: Classically activated (also defined M1 macrophages) and alternatively activated macrophages (also defined M2 macrophages or AAM), the former are proinflammatory, the latter anti-inflammatory [120]. It has been observed that IL4/IL13, two cytokines that induce a macrophage switch towards a M2 phenotype, are able to promote E-cadherin expression in macrophages, whereas IFNγ/LPS, triggering the development of M1 macrophages, produce the opposite effect [121,122]. This observation was corroborated by another study aimed at identifying an M2-specific gene signature. Using different mouse models of parasitic infections and cancer, pathologies associated to the development of M2 macrophages, the authors found that E-cadherin was one of the genes constituting the M2 signature, pointing to E-cadherin as M2 marker [123]. Moreover, E-cadherin in macrophages has a relevant role in granuloma formation [124,125,126]. For example, Cronan et al. observed that, during mycobacterial granuloma formation, macrophages undergo a process called mesenchymal–epithelial transition (MET) acquiring typical features of epithelial cells, including E-cadherin expression. Targeting E-cadherin led to granuloma disruption with the result of an increased immune clearance of the pathogen mediated by neutrophils and improved host survival, indicating that E-cadherin presence prevents an efficacious immune response [127]. In addition, E-cadherin overexpression in macrophages makes these cells hyporesponsive to proinflammatory stimuli, such TLR ligands, highlighting again the strict relationship between E-cadherin and the development of an immunosuppressive/immunotolerant environment [128]. Although it is not possible to draw any final conclusion based of the available data, it is possible to speculate that E-cadherin expression could be considered an indicator for DCs or macrophages characterized by a suppressive phenotype that in physiologic conditions is necessary to prevent uncontrolled and potentially harmful inflammatory processes but that can become detrimental in some pathologies, such as infectious diseases and cancer.

Moreover, E-cadherin not only can exert immune-modulatory functions in cells that express it, but also it can influence the behavior of other immune cells that possess receptors able to recognize this protein. For instance, it has been reported that E-cadherin can bind KLRG1 [129], an inhibitory receptor expressed by particular subsets of T cells and NK cells [130,131,132]. E-cadherin-KLRG1 interaction results in a signaling cascade leading to impairment of CD8^+^ T lymphocyte and NK cell proliferation and cytotoxicity that can be restored by treatment with antibodies able to disrupt KLRG1-E-cadherin binding [129,130,133]. Another partner of E-cadherin is represented by the heterodimer α_E_(CD103)β_7_ [134]. This protein complex can be expressed by immune cells, such as T lymphocytes, and is needed to allow the adhesion and the retention of these cells in different tissues for immunosurveillance [135]. The possibility for immune cells to be retained, for example, in the tumor microenvironment can be particularly advantageous for the host, since these cells can be activated in loco and exert effector function, eventually leading to the killing of tumor cells. Indeed, multiple reports indicate α_E_(CD103)β_7_ interaction with E-cadherin expressed by tumor cells promotes cytolytic T cell activity against different type of tumors such as lung [136] and pancreatic cancer [137]. These data may provide the biological explanation of several clinical data showing that high infiltration of CD8^+^ lymphocytes correlates with an improved survival in pancreatic cancer patients [138,139,140]. Moreover, it should be noted that CD103 marks also a particular subpopulation of DCs characterized by several functions (i.e., induction of regulatory T cells to maintain tolerance and antigen cross-presentation to CD8^+^ T cells), playing an important role in immune defense [141,142,143].

Although it is well recognized that E-cadherin does not merely represent a molecular glue able to maintain tissue integrity since it exerts a plethora of the most diverse functions, the implications of E-cadherin on immune system biology are not completely elucidated. To date, not many studies described the role of E-cadherin expression directly in immune cells. Even if we can assume that this protein can be associated with an immunotolerant/anti-inflammatory immune phenotype, this consideration cannot be always applied.

Since E-cadherin expressed by tumor cells is very important in determining PDAC fate, as described in detail in the previous paragraphs, it is legitimate to ask whether its expression on immune cells infiltrating pancreatic cancer may have the same relevance. However, it is difficult to address this question because of the paucity of information. However, we can try to make some speculations based on data obtained in studies on other tumor types or other diseases. First, E-cadherin may allow the adhesion of immune cells to the tumor mass, a situation resembling that occurring between Langerhans cells and keratinocytes in the epidermis [144,145]. The close proximity of the cells mediated by E-cadherin may be not only functional to stabilize the architecture of the tumoral tissue, but it may also create a bidirectional cross-talk able to influence the biology and behavior of the two partners of this molecular dialog, as described for Langerhans cells [146] and osteoclasts [147] where the interactions mediated by E-cadherin are required for the complete maturation/activation of these cells. Moreover, E-cadherin is also described to be one the proteins implicated in macrophage fusion [148] and in heterotypic interaction between macrophages and other cell types [118]. To this regard, Clawson et al. isolated and phenotypically characterized macrophage-tumor cell fusions (MTFs) from the blood of PDAC patients [149]. MTFs combine the properties of cancer cells and macrophages and can be a tool utilized by tumors to increase their invasiveness and metastatic potential [150,151]. Although not specifically mentioned, it is plausible to hypothesize the involvement of E-cadherin in the generation of MTFs. Second, PDAC is often characterized by lack of infiltration of CD8^+^ T lymphocytes [152] and, indeed, T cell infiltration is associated with PDAC patient survival [138,139,140]. Different factors can concur in limiting T cell trafficking in the tumor bed and macrophages are one of them [153,154,155]. For example, it has been reported that extratumoral macrophages negatively regulate the infiltration of T lymphocytes into PDAC [156] but less is known about TAMs, one the most abundant leukocytes infiltrating pancreatic cancer [157]. Peranzoni et al. observed that stromal macrophages hinder CD8^+^ T lymphocytes motility in lung cancer tumor microenvironment by cell–cell interaction [155]. A similar situation may likely occur also in PDAC. The mechanisms through which macrophages are able to exclude T cells from infiltrating tumor tissue are not very well defined but we can postulate the E-cadherin may participate in this phenomenon. As mentioned before, T cells can express α_E_(CD103)β_7_ [131] that, upon engagement with E-cadherin on macrophages, may determine a slowdown of lymphocytes. Moreover, TAMs, usually polarized towards an immunosuppressive M2-phenotype [157], can exploit the reduced spatial proximity with T cells to better inhibit their activity. However, the functional significance of this interaction may not be always detrimental. Indeed, as already mentioned, CD103^+^ T lymphocytes can recognize E-cadherin^+^ DCs and, in this way, they can be more easily activated [112].

Overall, these data suggest that E-cadherin can be considered also as an immunomodulator but, however, its role in the context of the immune response, especially against PDAC, is far to be completely understood and further investigations in the field are needed.

## 9. Conclusions

E-cadherin is expressed at a variable extent in PDAC tumors and cells allowing one to distinguish epithelial-like well differentiated or mesenchymal fibroblast-like phenotypes having a different invasive behavior. Moreover, partial EMT was frequently described in vivo and in vitro, so that PDAC cells can retain high E-cadherin expression at cell boundaries and possess functional adherens junctions although a highly invasive potential is evident.

The concomitant expression of epithelial-related phenotype and mesenchymal markers in the same cell indicate that the general assumption that an inverse correlation between E-cadherin expression and invasive potential of carcinoma cells is not absolute.

This apparent discrepancy opens some questions: how is it possible that PDAC cells exhibiting highly invasive and malignant behavior retain a differentiated epithelial phenotype? What is the role of E-cadherin in these cells? Do these cells undergo EMT?

To find an answer, we should consider the overall characteristics of EMT and PDAC cells. The first point to be considered is that EMT is not an “all or nothing” phenomenon but rather a multistep process consisting of a broad range of phenotypic changes. Moreover, the different steps do not occur consecutively and not all of them necessarily occur. This characteristic of the EMT mechanism can be responsible of the concomitant expression of epithelial and mesenchymal markers and, therefore, of the partial EMT-related phenotype observed in PDAC cells.

**Figure 4:**
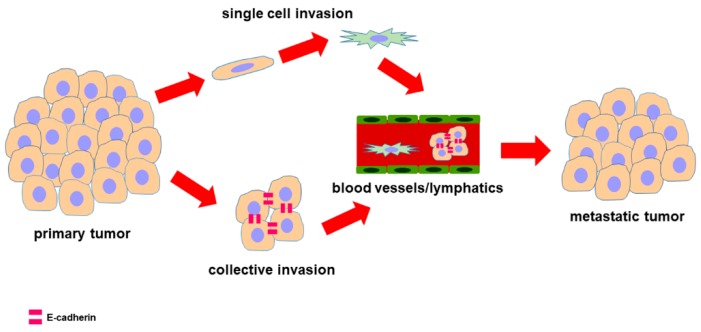
Diagram showing the possible strategies used by carcinoma cells for migration and invasion. Carcinoma cells, having a differentiated epithelial phenotype, retain E-cadherin at the cell membrane providing a strong cohesion during collective migration and invasion. By contrast, single cell invasion is played by cells that, after losing their epithelial features during EMT, switched to a mesenchymal phenotype. Once arrived at metastatic niche, these cells reacquired epithelial characteristics through another process defined mesenchymal to epithelial transition (MET).

Second point, it was demonstrated that some PDAC cells have a highly invasive and malignant behavior although they retain a differentiated epithelial phenotype and E-cadherin expression at the plasma membrane. This morphological characteristic does not exclude the invasive behavior since tumor cells can invade the surrounding tissues adopting different morphology and migration patterns (Figure 4). In fact, mesenchymal fibroblast-like cells move individually after detaching from the tumor, but, interestingly collective migration of multicellular units is also possible as described in breast, prostate, large cell lung and ovarian cancers, including PDAC [49,158,159,160,161,162]. Functional adherens junctions containing E-cadherin are needed to provide PDAC cells with higher cell adhesion and to ensure tissue integrity, thus favoring collective cell migration.

The third and last consideration is referred to the in vivo evaluation of E-cadherin in human PDAC specimens. When analyzing PDAC cells in vitro, the detection of EMT markers and their relationship in determining cell phenotype can be easily deciphered than in tumor samples. In tumor tissues, E-cadherin is frequently retained and its loss undetectable, so that there is no clear demonstration of EMT in human tumor biopsies. In relation to this issue, we have to consider that most human tumors are characterized by cellular heterogeneity, so that a clear phenotype does not prevail. Moreover, even if E-cadherin is retained, we cannot exclude that cells underwent EMT, since this process might be highly localized and transient, and possibly functions as a brief proinvasion conversion program limited to specific steps and timing in metastatic colonization.

**Figure 5:**
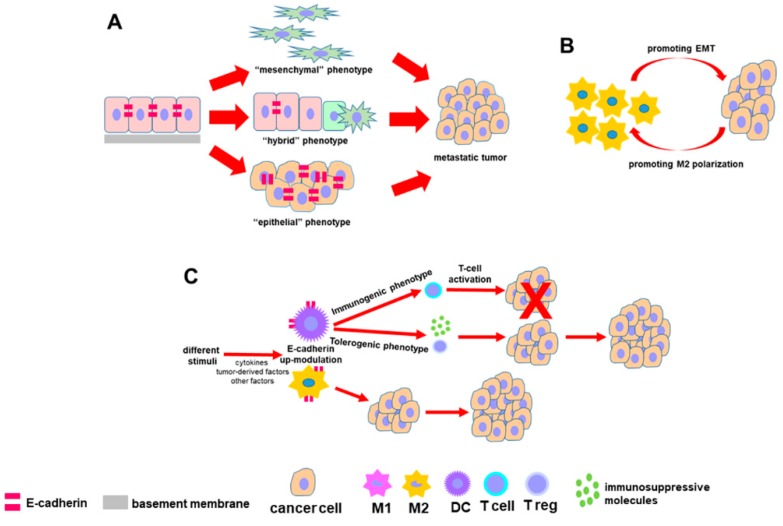
Diagram summarizing the relationship between E-cadherin expression and the key events of EMT. The involvement of E-cadherin and its interplay within the immune cells in tumor microenvironment possibly influencing the PDAC cell fate is also included. (**A**) E-cadherin differently influences the behavior of cancer cells having a different EMT-related cell phenotype. (**B**) Macrophages and pancreatic tumor cells establish a bidirectional cross-talk: The former promotes EMT in cancer cells and, in turn, the latter influences macrophage polarization toward a M2 phenotype. (**C**) In DCs, E-cadherin expression can be associated either with an immunogenic phenotype able to promote an efficacious immune response against tumor, either with a tolerogenic phenotype that suppresses the immune response supporting tumor growth. In macrophages, E-cadherin up-regulation is usually correlated with a M2 protumor phenotype.

Since E-cadherin is the major determinant of the epithelial phenotype and the main actor of the EMT process, the characterization of E-cadherin multifaceted expression allows the interpretation of different roles during the steps of EMT (Figure 5). Only the deciphering of its expression in relation to the cell phenotype and the timing of its loss during the transition of normal ductal epithelium to invasive cancer will allow to better understand PDAC biology and behavior, in order to develop new and effective therapeutic tools.
